# Integrative analysis of metabolomics and proteomics reveals amino acid metabolism disorder in sepsis

**DOI:** 10.1186/s12967-022-03320-y

**Published:** 2022-03-14

**Authors:** Qi Chen, Xi Liang, Tianzhou Wu, Jing Jiang, Yongpo Jiang, Sheng Zhang, Yanyun Ruan, Huaping Zhang, Chao Zhang, Peng Chen, Yuhang Lv, Jiaojiao Xin, Dongyan Shi, Xin Chen, Jun Li, Yinghe Xu

**Affiliations:** 1 Precision Medicine Center, Taizhou Central Hospital (Taizhou University Hospital), Taizhou, China; 2 State Key Laboratory for Diagnosis and Treatment of Infectious Diseases, Collaborative National Clinical Research Center for Infectious Diseases, The First Affiliated Hospital, Zhejiang University School of Medicine, Hangzhou, China; 3 Key Laboratory for Critical Care Medicine, Department of Critical Care Medicine, Taizhou Central Hospital (Taizhou University Hospital), Taizhou, China; 4 Department of Intensive Care Unit, Taizhou Hospital of Zhejiang Province, Taizhou Central Hospital (Taizhou University Hospital), Wenzhou Medical University, 999 Donghai Avenue, Taizhou, 318000 China; 5 Department of Intensive Care Unit, Taizhou Enze Medical Center (Group) Enze Hospital, Taizhou, China; 6 Institute of Pharmaceutical Biotechnology and the First Affiliated Hospital Department of Radiation Oncology, Zhejiang University School of Medicine, Hangzhou, 310058 China; 7 Joint Institute for Genetics and Genome Medicine Between, Zhejiang University and University of Toronto, Zhejiang University, Hangzhou, China

**Keywords:** Sepsis, Multiomics, Biomarker, Untargeted metabolomics, Proteomics

## Abstract

**Background:**

Sepsis is defined as a systemic inflammatory response to microbial infections with multiple organ dysfunction. This study analysed untargeted metabolomics combined with proteomics of serum from patients with sepsis to reveal the underlying pathological mechanisms involved in sepsis.

**Methods:**

A total of 63 patients with sepsis and 43 normal controls were enrolled from a prospective multicentre cohort. The biological functions of the metabolome were assessed by coexpression network analysis. A molecular network based on metabolomics and proteomics data was constructed to investigate the key molecules.

**Results:**

Untargeted metabolomics analysis revealed widespread dysregulation of amino acid metabolism, which regulates inflammation and immunity, in patients with sepsis. Seventy-three differentially expressed metabolites (|log_2_ fold change| > 1.5, adjusted *P* value < 0.05 and variable importance in the projection (VIP) > 1.5) that could predict sepsis were identified. External validation of the hub metabolites was consistent with the derivation results (area under the receiver operating characteristic curve (AUROC): 0.81–0.96/0.62–1.00). The pentose phosphate pathway was found to be related to sepsis-associated encephalopathy. Phenylalanine metabolism was associated with sepsis-associated acute kidney injury. The key molecular alterations of the multiomics network in sepsis compared to normal controls implicate acute inflammatory response, platelet degranulation, myeloid cell activation involved in immune response and phenylalanine, tyrosine and tryptophan biosynthesis, and arginine biosynthesis.

**Conclusions:**

Integrated analysis of untargeted metabolomics and proteomics revealed characteristic metabolite and protein alterations in sepsis, which were mainly involved in inflammation-related pathways and amino acid metabolism. This study depicted the pathological characteristics and pathways involved in sepsis and potential therapeutic targets.

**Supplementary Information:**

The online version contains supplementary material available at 10.1186/s12967-022-03320-y.

## Background

Sepsis is a complicated life-threatening syndrome with organ dysfunction caused by the maladjustment of the host response to infection [1]. A study in 2020 showed that there were 48.9 million sepsis patients worldwide, resulting in 11 million deaths and accounting for 19.7% of the total death toll [2]. The WHO identified sepsis as a global health priority and urged countries to reduce the global burden of sepsis [3]. At present, the standard treatment for sepsis is early resuscitation, infection focus clearance, use of antibiotics and organ support therapy. However, although there have been many advances in treatment strategies for sepsis in recent years, the mortality rate of sepsis remains high. The reason is that the pathophysiology involved in sepsis progression remains unclear. There is an urgent need to accurately define sepsis, reveal its complex molecular characteristics, and establish technology for the accurate detection of multiple molecular markers for early diagnosis, early warning and prediction of sepsis to ensure that patients can receive accurate and effective intervention in a timely manner.

Metabolomics is a research method of qualitatively and quantitatively analysing all the metabolites of small molecules in organisms and identifying the relationship between metabolites and physiological and pathological changes [4]. Most of the analytes are small molecules with molecular weights of less than 1500 Da that can be used as important indicators of physiological or pathological states and help to understand the occurrence and progression of diseases [5]. Several studies have used metabolomics analysis to identify novel biomarkers associated with the disease progression, mechanism and prognosis of sepsis and found distinct metabolic profiles in sepsis [6–8]. These studies used only metabolomics techniques with differential analysis and pathway enrichment analysis to illustrate the metabolic disorder underlying the mechanism in sepsis. Identification of coexpression patterns based on multiomics data might reveal new insights into the mechanism of sepsis. Weighted gene coexpression network analysis (WGCNA) is a topological algorithm that can be used to investigate the relationship between omics data and clinical phenotypes and has been widely used in transcriptomics, proteomics and metabolomics studies [9]. With the development of multiomics detection and analysis technology, the establishment of an accurate detection system for multiple molecular markers for early diagnosis, early warning and prediction of sepsis based on evidence-based medicine and big multiomics data would be a strategic breakthrough [10]. Integrative multiomics analysis could provide valuable insight into biological functions with mutual validation, which might not be revealed in a single dataset.

In this study, metabolomics was used to measure the aggregation of all metabolized small molecular components in sepsis. WGCNA and integrative multiomics analysis of the same biological samples were performed to study the changing rules of metabolites, reveal the metabolic nature of sepsis, and identify the metabolic micromolecular features or markers of sepsis diagnosis and pathogenesis.

## Methods

### Study design

**Fig. 1:**
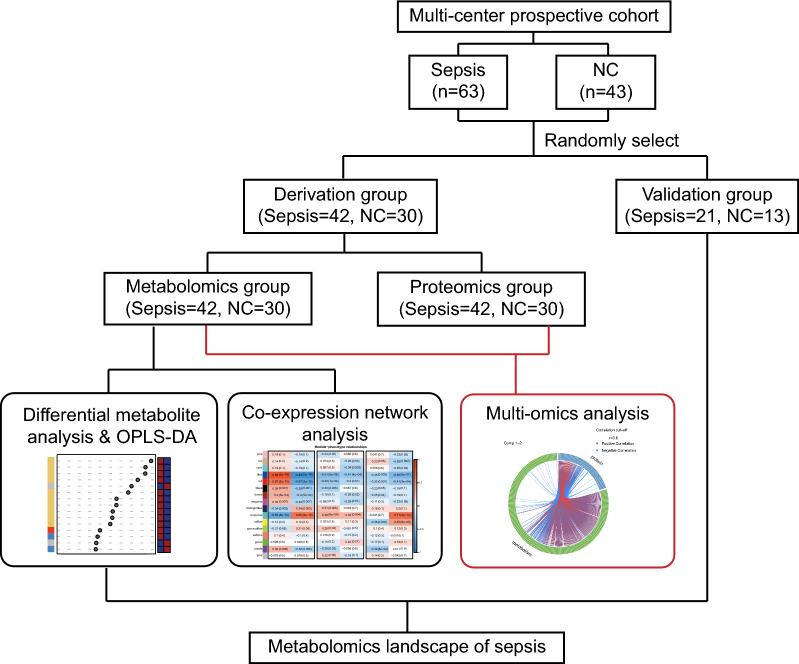
Overview of the study design and patient group allocation. One hundred six subjects were studied, of whom 72 were randomly selected for proteomic and metabolomic analysis, and 34 were the validation group. *NC* normal control

In this study, patients with sepsis were enrolled from three intensive care units (ICUs) from April 1, 2019, to August 16, 2020. The clinical and follow-up data were collected from the electronic data capture system and case report forms. The final follow-up was completed on November 20, 2020. Patients were diagnosed with sepsis according to an acute change in the total Sequential Organ Failure Assessment (SOFA) score of 2 or more points due to an infection [1]. At the same time, healthy normal control (NC) subjects (with a SOFA score of 0 and no infection) were recruited as the control group from the Physical Examination Center. The patients and NC subjects were randomly allocated into the derivation and validation groups (Fig. 1). Differential expression analysis and coexpression network analysis were performed to identify variations in metabolites and pathways associated with the clinical pathophysiology of sepsis. Integrative analysis combining metabolomics and proteomics data of the same biological samples was conducted to acquire the comprehensive landscape of sepsis. The study protocol was approved by the Clinical Research Ethics Committee of Taizhou Central Hospital (Taizhou University Hospital) (Registration Number: 2019–016; principal investigator: Yinghe Xu; date of registration: 26 February 2019). Written informed consent was obtained from all participants or their legal representatives.

### Sample collection and preparation for metabolomic analysis

Blood samples were collected from patients diagnosed with sepsis at admission to the ICU. Blood samples of patients and NC subjects were drawn using serum separation tubes and allowed to clot at room temperature for 60 min. The samples were centrifuged for 10 min within 30 min (1600×*g*, 4 °C) to remove insoluble solids. Each aliquot of serum was collected and immediately stored at − 80 °C until ultrahigh-performance liquid chromatography with quadrupole time-of-flight mass spectrometry (UPLC-Q-TOF/MS) analysis.

### Metabolomic LC–MS/MS analysis

Metabolomic LC–MS/MS analysis was performed using ultrahigh-performance liquid chromatography (1290 Infinity LC, Agilent Technologies) coupled to a quadrupole time-of-flight instrument (AB Sciex TripleTOF 6600, Shanghai Applied Protein Technology Co., Ltd).

For hydrophilic interaction liquid chromatography (HILIC) separation, a 2.1 mm × 100 mm ACQUIY UPLC BEH 1.7 μm column (Waters, Ireland) was used to analyse the samples. In both positive and negative ion-mode electrospray ionization (ESI), the mobile solutions consisted of 25 mM ammonium acetate, 25 mM ammonium hydroxide in water and acetonitrile. The gradient was 85% acetonitrile for 1 min and was then linearly decreased to 65% in 11 min, reduced to 40% in 0.1 min, maintained for 4 min, increased to 85% in 0.1 min and maintained for 5 min.

For reversed-phase liquid chromatography (RPLC) separation, a 2.1 mm × 100 mm ACQUIY UPLC HSS T3 1.8 μm column (Waters, Ireland) was used to analyse the samples. In ESI positive ion mode, the mobile solutions consisted of water with 0.1% formic acid and acetonitrile with 0.1% formic acid. In ESI negative ion mode, the mobile phase consisted of 0.5 mM ammonium fluoride in water and acetonitrile. The gradient was 1% acetonitrile for 1.5 min and was gradually increased to 99% in 11.5 min and maintained for 3.5 min. Then, it was reduced to 1% in 0.1 min, and a 3.4 min re-equilibration period was employed. The gradients had a flow rate of 0.3 mL/min, and the column temperatures were kept constant at 25 °C. A 2 μL aliquot of each sample was injected.

The ESI source conditions were set as follows: ion source gas 1 at 60 psi, ion source gas 2 at 60 psi, curtain gas at 30 psi, source temperature: 600 °C, and ion spray voltage floating ± 5500 V. In MS-only acquisition, the instrument was set to acquire over an m/z range of 60–1000 Da, and the accumulation time for the TOF MS scan was set at 0.20 s/spectra. In auto MS/MS acquisition, the instrument was set to acquire over an m/z range of 25–1000 Da, and the accumulation time for the product ion scan was set at 0.05 s/spectra. The product ion scan was acquired using information-dependent acquisition with high sensitivity mode selected. The parameters were set as follows: the collision energy was fixed at 35 V with ± 15 eV; declustering potential, 60 V (+) and − 60 V ( −); exclusion of isotopes within 4 Da; and candidate ions to monitor per cycle: 10.

### Metabolomic data processing

ProteoWizard MSConvert was applied to convert the raw MS data (wiff.scan files) to MzXML files. XCMS software was used for peak alignment, retention time correction and peak area extraction. For peak finding, the parameters were set as follows: centWave m/z = 25 ppm, peakwidth = c (10, 60), prefilter = c (10, 100). For peak grouping, the parameters were set as follows: bw = 5, mzwid = 0.025, minfrac = 0.5. For peak annotation, Collection of Algorithms of MEtabolite pRofile Annotation was used to annotate the isotopes and adducts. The extracted ion features with more than 50% of the nonzero measurement values in at least one group were included in further analysis. The identification of metabolites met the following criteria: narrow window retention index, accurate m/z value with variation less than 25 ppm and MS/MS spectra with high scores based on comparisons with the in-house database (Shanghai Applied Protein Technology) established with available authentic standards [11, 12]. The identified metabolites met the level 2 and above requirements by the Chemical Analysis Working Group of the Metabolomics Standards Initiative. Open database sources, including the Human Metabolome Database, Kyoto Encyclopedia of Genes and Genomes (KEGG) pathway database and MetaboAnalyst, were used to identify metabolic pathways.

### Serum proteomics data

Proteomics analysis has been described previously [13] and used the same biological samples as metabolomics. All proteomic data from the patients with sepsis and NCs analysed in this study were obtained from the ProteomeXchange Consortium (http://proteomecentral.proteomexchange.org) via the iProX partner repository with the dataset identifier IPX0003225000.

### Metabolite coexpression network analysis

WGCNA was performed to identify metabolite coexpression modules based on untargeted metabolomics data [14]. The dimensions of the dataset were reduced by clustering highly correlated metabolites into modules, and the module membership measure was defined as kME. The correlation between the module and sample clinical traits was calculated to identify metabolite modules that were highly associated with the clinical phenotype of interest. The function WGCNA:blockwiseModules() was applied to construct the coexpression network with the following settings: soft threshold power β = 3 (calculated based on the scaled-free topology model parameters), deepSplit = 4, minModuleSize = 10, mergeCutHeight = 0.05, threshPercent = 50, and mergePercent = 25; all other parameters were set to the default.

### Multiomics data integration

To integrate the analysis of metabolomic and proteomic data, sparse generalized canonical correlation discriminant analysis via Data Integration Analysis for Biomarker Discovery using Latent cOmponents (DIABLO) [15] in the R package mixOmics [16] was performed. The method applied the generalized, supervised partial least-squares approach to integrate multiple data types across the same biological samples and jointly identify key omics features across multiple datasets. Normalized metabolomic and proteomic data were log-transformed before integration by DAIBLO.

### Statistical analysis

The measurement results are presented as numbers (percentages) for categorical variables, the mean ± standard deviation (SD) for normally distributed variables, and the median with interquartile range (IQR) for nonnormally distributed variables, unless indicated otherwise. Welch’s t test was applied to compare two continuous variables when each compared group was normally distributed (Shapiro–Wilk test when *P* value > 0.05). The nonparametric Mann–Whitney U test was applied to compare the nonnormally distributed data between groups. The false discovery rate with the Benjamini–Hochberg procedure was used to control type I error in multiple tests. The extracted ion features with more than 50% of the nonzero measurement values were included in further analysis. Missing values were imputed with minimal values in the metabolomics dataset. Log10 transformation of the metabolite abundance was performed after sum-normalization. The χ^2^ test was used to compare categorical variables. Statistical significance and differentially expressed metabolites (DEMs) were defined as “log_2_ fold change (FC) > 1.5, adjusted *P* value < 0.05”.

Statistical analysis was performed using R software unless noted otherwise. Principal component analysis (PCA) was performed by using the “PCA” function in the FactoMineR package. Orthogonal partial least-squares discrimination analysis (OPLS-DA), pathway analysis and visualization were performed by using MetaboAnalyst 5.0 (http://www.metaboanalyst.ca/MetaboAnalyst/).

## Results

### Patients and clinical characteristics

A total of 63 patients with sepsis and 43 NC subjects were enrolled in the study and randomly allocated to the derivation group (42 patients with sepsis and 30 NCs) and validation group (21 patients with sepsis and 13 NCs) (Fig. 1). The clinical characteristics of patients with sepsis in the derivation and validation groups are provided in Table 1. The disease severity was consistent between these two groups, of which the SOFA scores were 6.0 [4.0, 9.0] and 8.0 [6.0, 10.0], and the Acute Physiology and Chronic Health Evaluation (APACHE) II scores were 19.0 [10.2, 22.7] and 22.0 [18.0, 25.0], respectively. The short-term (28/90 day) mortality was 14.3%/14.3% in the two groups. Most patients had a gram-negative bacterial infection (42.9% and 33.3%, respectively), and some patients had a gram-positive bacterial infection (9.5% and 19.0%, respectively). Age, sex and clinical laboratory indices were not significantly different between these two groups (*P* > 0.05). The clinical characteristics of patients with sepsis and NC subjects in the derivation group are summarized in Additional file 2: Table S1. Age and sex were not different between the patients and NCs (*P* > 0.05).

**Table 1:** Characteristics of enrolled patients with sepsis included in the derivation group and validation group

	Derivation group	Validation group	*P* value
n	42	21	
Male (%)	26 (61.9%)	14 (66.7%)	0.926
Age (years)	71.5 [61.0, 78.0]	70.00 [63.0, 79.0]	0.743
Laboratory data			
Mean arterial pressure (mm Hg)	79.7 [74.0, 89.0]	76.3 [62.7, 90.7]	0.635
White blood cell count (10^9^/L)	14.3 [10.1, 21.1]	14.00 [9.4, 23.5]	0.931
Haemoglobin (g/L)	119.0 [101.5, 129.5]	103.0 [87.0, 118.0]	0.027
Haematocrit (%)	35.8 [31.5, 38.8]	31.6 [27.0, 37.4]	0.084
Platelet count (10^9^/L)	136.0 [60.7, 209.5]	118.0 [71.0, 202.0]	0.849
Albumin (g/dL)	27.0 [24.4, 30.9]	24.90 [23.2, 28.0]	0.251
Aspartate aminotransferase (U/L)	45.0 [21.2, 89.5]	33.0 [15.0, 127.0]	1.000
Alanine aminotransferase (U/L)	29.5 [14.5, 61.5]	25.0 [17.0, 74.0]	0.905
Total bilirubin (μmol/L)	16.5 [9.0, 25.1]	11.1 [8.4, 20.4]	0.424
Creatinine (μmol/L)	133.0 [98.7, 226.0]	163.0 [104.0, 229.0]	0.553
INR	1.2 [1.1, 1.3]	1.2 [1.1, 1.4]	0.951
Infection			0.661
Gram-positive bacteria (%)	4 (9.5%)	4 (19.0%)	
Gram-negative bacteria (%)	18 (42.9%)	7 (33.3%)	
Viral (%)	1 (2.4%)	0 (0.0%)	
Other (%)	19 (45.2%)	10 (47.7%)	
CRRT	8 (13.6%)	NA	NA
Vasopressors			0.124
0 (%)	18 (42.9%)	6 (28.6%)	
1 (%)	20 (47.6%)	15 (71.4%)	
NA (%)	4 (9.5%)	0 (0.0%)	
Mechanical ventilation			0.190
0 (%)	21 (50.0%)	12 (57.1%)	
1 (%)	15 (35.7%)	9 (42.9%)	
NA (%)	6 (14.3%)	0 (0.0%)	
Severity at time of admission to ICU			
SOFA	6.0 [4.0, 9.0]	8.0 [6.0, 10.0]	0.115
APACHE II	19.0 [10.2, 22.7]	22.0 [18.0, 25.0]	0.074
Mortality			
28-day	6 (14.3%)	3 (14.3%)	1
90-day	6 (14.3%)	3 (14.3%)	1

Data are expressed as the mean ± SD, median (IQR) or number of patients (percentages). Continuous variables were compared by using Student’s t test and the Mann–Whitney U test, and categorical variables were compared by using the χ^2^ or Fisher’s exact test between the derivation and validation groups

*APACHE II* acute physiology and chronic health evaluation II, *SOFA* sequential organ failure assessment on day of sampling

### Untargeted metabolomic profiling of serum from patients with sepsis

To ensure the reliability of untargeted metabolomic data, the total ion chromatogram of quality control (QC) samples showed that the response intensity and retention time of each chromatographic peak basically overlapped, indicating the stability of the instrument (Additional file 1: Figure S1A). The PCA plot showed that QC samples closely clustered in positive and negative ion modes, demonstrating the good repeatability of the LC–MS analysis during the experiment. (Additional file 1: Figure S1B).

**Fig. 2:**
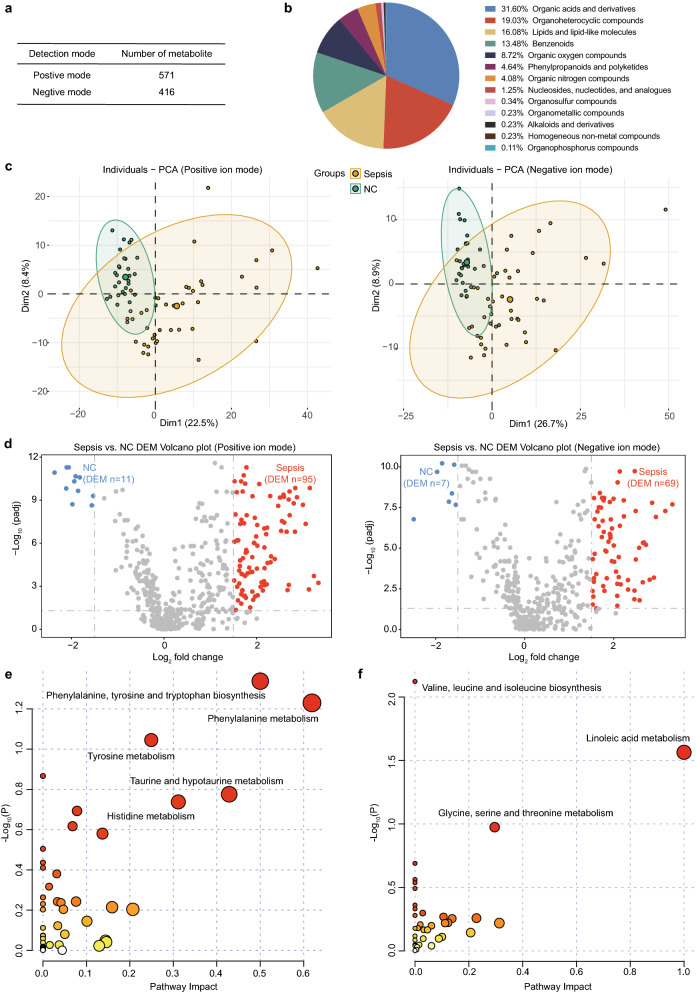
Comprehensive analysis of untargeted metabolomics of serum from patients with sepsis and normal controls. **A** The number of metabolites detected in positive and negative ion modes. **B** The proportion of the identified metabolites in each chemical classification. **C** Principal component analysis (PCA) of the identified metabolites showing that patients with sepsis differed from normal controls (NC) in both positive ion mode (left) and negative ion mode (right). **D** The volcano plot of differentially expressed metabolites (DEMs) between patients with sepsis and NCs in positive ion mode (left) and negative ion mode (right). The metabolites whose expression increased in sepsis are shown in red and those whose expression decreased are shown in blue. **E**–**F** Pathway analysis of upregulated and downregulated metabolites

A total of 987 metabolites (Fig. 2A), including organic acids and derivatives, organoheterocyclic compounds, lipids, benzenoids and others (Fig. 2B), were identified. The PCA plot based on the identified untargeted metabolites showed that patients with sepsis were different from NC subjects in both the positive and negative ion modes (Fig. 2C). Differential expression analysis with the cut-off of a Benjamini–Hochberg adjusted filter of < 0.05 and log_2_ FC > 1.5 showed that 106 (95 upregulated and 11 downregulated) and 76 (69 upregulated and 7 downregulated) metabolites were significantly changed in the patients with sepsis in positive and negative ion modes, respectively (Fig. 2D). Further details of the DEMs are shown in Additional file 3: Table S2 and Additional file 4: Table S3. Pathway analysis of the upregulated metabolites revealed a combination of metabolites from positive and negative ion modes that were most enriched in amino acid metabolism, such as phenylalanine, tyrosine and tryptophan biosynthesis, phenylalanine metabolism, tyrosine metabolism, etc. (Fig. 2E). The downregulated metabolites in sepsis mainly participated in valine, leucine and isoleucine biosynthesis, linoleic acid metabolism and glycine, serine and threonine metabolism (Fig. 2F).

### Candidate biomarker metabolites for diagnosing patients with sepsis

**Fig. 3:**
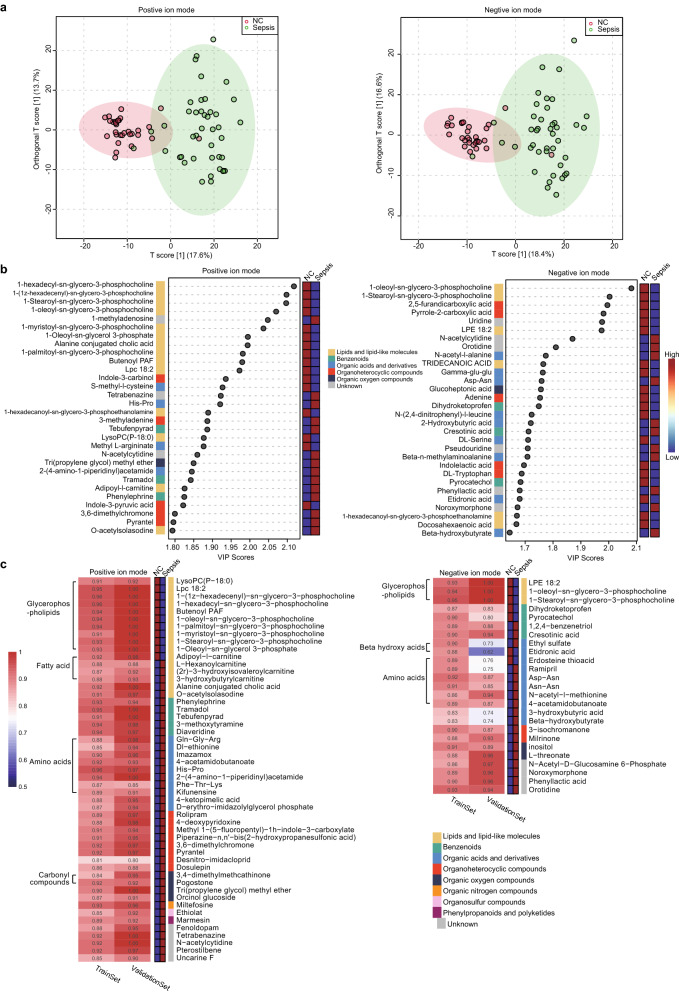
Dysregulated metabolites in patients with sepsis. **A** Orthogonal partial least-squares discriminant analysis (OPLS-DA) of serum metabolism showing that patients with sepsis substantially differed from normal controls (NCs) in both positive (left) and negative ion modes (right). **B** The top 30 metabolites with high discriminatory accuracy ranked by variable importance in projection (VIP) score in positive ion mode (left) and negative ion mode (right). **C** The area under the receiver operating characteristic curve (AUROC) to assess the discriminatory accuracy of 51 metabolites (left) and 25 metabolites (right) in differentiating patients with sepsis from normal controls in the derivation and validation groups

OPLS-DA analysis showed a substantial separation between patients with sepsis and NC subjects in both the positive and negative ion modes, indicating that there were obvious differences in their serum untargeted metabolomic profiles (Fig. 3A). Permutation analysis validated the OPLS-DA model with R^2^Y = 0.776 and Q^2^ = 0.647 for positive ion mode and R^2^Y = 0.753 and Q^2^ = 0.651 for negative ion mode (Additional file 1: Figure S2). The top 30 metabolites with high variable importance in the projection (VIP) scores are shown in Fig. 3B, illustrating that they have great potential to distinguish patients with sepsis from NC subjects. To explore the candidate biomarker metabolites that could diagnose patients with sepsis, 51 and 25 metabolites that were significant DEMs (log_2_ FC > 1.5 & adjusted *P* value < 0.05) and had VIP scores > 1.5 were identified in positive and negative ion modes, respectively (Additional file 1: Figure S3). The area under the receiver operating characteristic curve (AUROC) was calculated to assess the discriminatory accuracy of candidate biomarker metabolites. The results showed that the AUROCs of 73 metabolites, comprising the 51 and 25 metabolites indicated above, ranged from 0.81 to 0.96 (Fig. 3C). The validation group exhibited consistent results, of which the AUROC ranged from 0.62 to 1.00. Among the 73 metabolites, some fatty acids, such as 3-hydroxybutyrylcarnitine and l-hexanoylcarnitine, have functions in energy production, and glycerophospholipids have a vital role in lipid signal transduction.

### Construction of a sepsis metabolite coexpression network

**Fig. 4:**
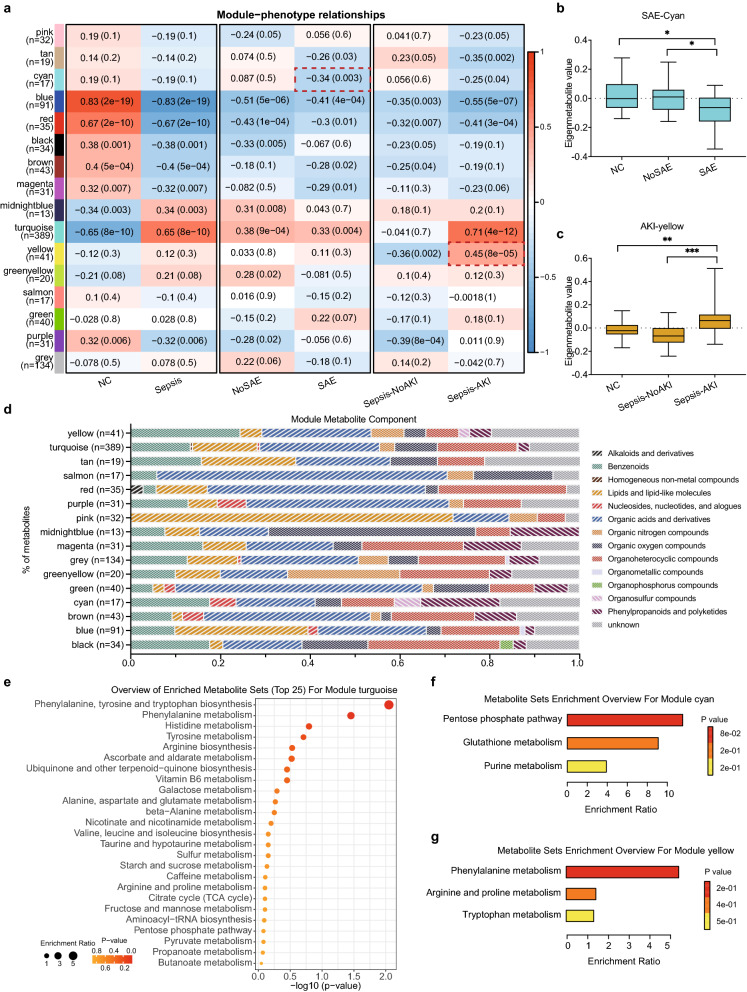
The coexpression network of serum metabolites constructed by weighted gene coexpression network analysis. **A** Heatmap representation of the correlation between module eigenmetabolites and different phenotypes of normal control (NC) and sepsis (left), sepsis-associated encephalopathy (SAE) (middle), and sepsis-associated acute kidney injury (Sepsis-AKI) (right). **B** Synthetic eigenmetabolite analysis of the module cyan module, which is highly correlated with SAE, except the module turquoise. **C** Synthetic eigenmetabolite analysis for module yellow, which is highly correlated with sepsis-AKI, except for module turquoise. **D** The distribution of metabolite members in each module according to the metabolite categories in Fig. 1B. **E** Pathway analysis of metabolites in module turquoise. **F** Pathway analysis of metabolites in module cyan. **G** Pathway analysis of metabolites in module yellow

A metabolite coexpression network was constructed using WGCNA based on the correlation patterns among the identified metabolites. A total of 16 metabolite modules with high coexpression were identified (Additional file 1: Figure S4). The size of the modules ranged from 13 metabolites (module midnight blue) to 389 metabolites (module turquoise). Correlation analysis between each metabolite module and the disease phenotype was performed to identify the modules that were significantly associated with the clinical phenotypes of interest. The results showed that module turquoise was highly correlated with sepsis (R = 0.65, *P* value = 8e−10) (Fig. 4A). In addition to module turquoise, module cyan was found to be relevant to sepsis-associated encephalopathy (SAE, R =  − 0.34, *P* value = 0.003), and module yellow was correlated with sepsis-associated acute kidney injury (sepsis-AKI, R = 0.45, *P* value = 8e−10). The eigenmetabolite value of module cyan significantly decreased across the NC, NoSAE and SAE phenotypes (Fig. 4B). Similarly, the eigenmetabolite value of module yellow significantly increased across the NC, Sepsis-NoAKI and Sepsis-AKI phenotypes (Fig. 4C). The categories of metabolites in each module are shown in Fig. 4D. Benzenoids, organic acids and their derivatives were the majority of metabolite members of the turquoise, cyan and yellow modules. Lipids and lipid-like molecules were present in the turquoise and yellow modules but not in module cyan, which included metabolites of phenylpropanoids and polyketides. Pathway analysis showed that the metabolites of module turquoise were mainly enriched in phenylalanine, tyrosine and tryptophan biosynthesis, phenylalanine metabolism, and histidine metabolism, etc. (Fig. 4E). Metabolites of module cyan, which was negatively correlated with SAE, were enriched with the pentose phosphate pathway (Fig. 4F). The metabolites that were positively associated with sepsis-AKI in module yellow played a part in phenylalanine metabolism (Fig. 4G).

### Integrative analysis of untargeted metabolomic and proteomic data

**Fig. 5:**
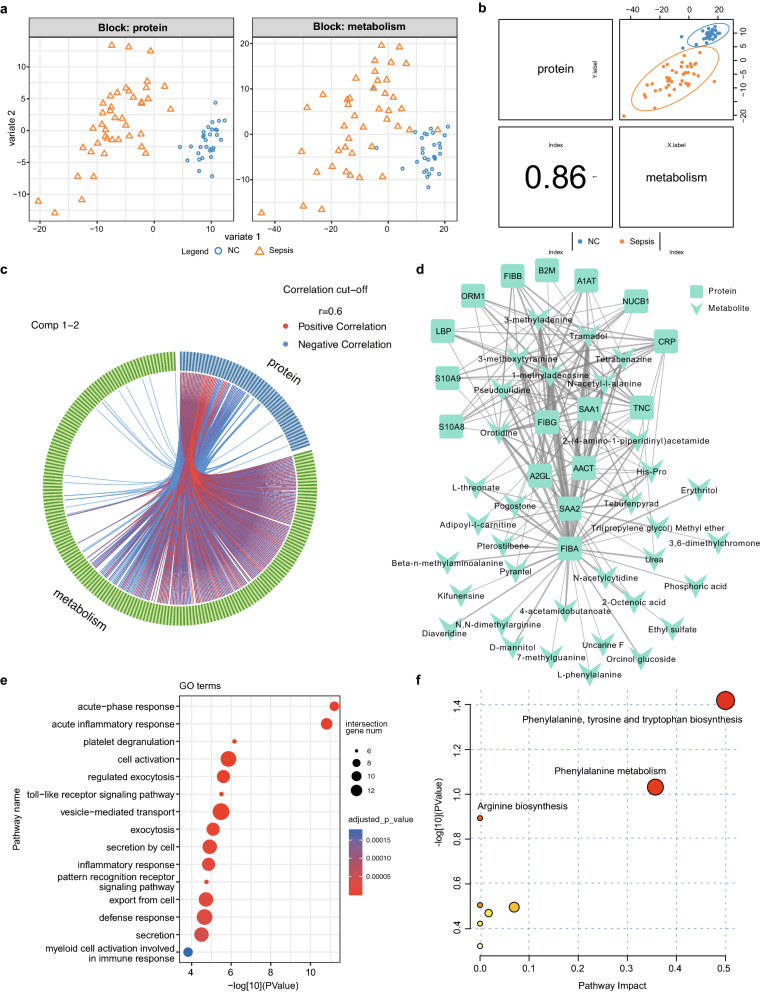
Integrative network analysis of proteomics and untargeted metabolomics data. **A** The considerable discrimination between patients with sepsis and normal controls (NC) in both proteomics (left) and untargeted metabolomics (right) data. **B** The Pearson correlation between proteomics and untargeted metabolomics data of their first component. **C** Circos plot of close correlations (Pearson coefficient cut-offs set at ≥ 0.6 or ≤  − 0.6) between proteomics and untargeted metabolomics data. **D** The network of key features across proteomics and untargeted metabolomics. The thickness of the edge represents the correlation coefficient. **E** GO terms enriched by the key proteins. **F** Pathway analysis of the key metabolites

To construct a comprehensive profile of sepsis and identify the relationships between metabolites and proteins, multiomics analysis integrating proteomic and untargeted metabolic data based on the same biological samples was performed. The sample plot of the DIABLO model showed that patients with sepsis were significantly different from NC subjects in both the proteomic and untargeted metabolomic data (Fig. 5A). The latent components of the two omics datasets were highly correlated, illustrating that the DIABLO model of the two datasets had good agreement (Fig. 5B). A significantly positive and negative correlation between proteins and metabolites were identified (Fig. 5C). A cluster of coregulated features strongly relevant to the latent components of the multiomics dataset was identified, which might be a potential characteristic of sepsis (Fig. 5D). Functional enrichment analysis revealed that proteins of the coregulated features were mainly involved in the acute inflammatory response, Toll − like receptor signalling pathway, defense response and myeloid cell activation involved in immune response (Fig. 5E). Metabolites were mostly enriched in amino acid metabolism, such as phenylalanine, tyrosine and tryptophan biosynthesis and phenylalanine metabolism, which has an important function in adaptive and innate immunity (Fig. 5F). These results indicated a potential regulatory crosstalk of inflammatory response and amino acid metabolism, which might provide a viable approach to suppress the systemic inflammatory response syndrome of sepsis.

## Discussion

Sepsis is a life-threatening organ dysfunction syndrome caused by a dysregulated host response to infection [17]. Sepsis has been found to be associated with metabolic alterations [18]. Elucidating the pathophysiological characteristics of sepsis could help to reduce its high morbidity and mortality. In this study, we analysed untargeted metabolomics data in blood samples to unveil the alteration of metabolites in sepsis. Combined multiomics analysis of metabolomics and proteomics data based on the same biological samples was performed to construct a reliable network for system biology analysis of sepsis from protein to the final metabolic product and identify the signatures associated with the characteristics of sepsis as changes in proteins and metabolites.

**Fig. 6:**
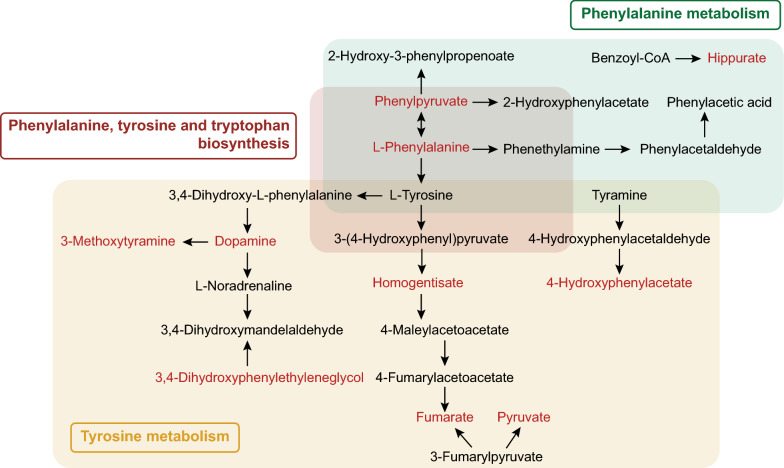
Schematic diagram of the crucial pathways. Phenylalanine, tyrosine and tryptophan biosynthesis, phenylalanine metabolism and tyrosine metabolism were the top hits from the pathway analysis of sepsis-specific metabolites. The upregulated metabolites in sepsis are coloured red

Amino acid pathways have been found to be dysregulated in bacterial infection [19]. Studies have reported that amino acid metabolism plays an essential role in adaptive and innate immunity, regulating the activation of immune cells and the production of antibodies [20]. In this study, amino acid metabolism, including phenylalanine, tyrosine and tryptophan biosynthesis, phenylalanine metabolism, and histidine metabolism, was observed to be the most marked and widespread alteration in sepsis. The occurrence of metabolic disturbances along with variations in amino acid levels has been found in septic patients and might have a function in the pathogenesis of septic encephalopathy [21]. Amino acid sensing is related to controlling intestinal inflammation [22]. Changes in amino acid consumption and the downstream molecular pathways have generally become a field of interest in generating novel drugs and therapeutic targets that might control immunity [23]. The essential intermediary metabolites in the aromatic amino acid-related (phenylalanine, tyrosine and tryptophan) pathways were found to have changed in sepsis (Fig. 6). We observed that most aromatic amino acid-related pathway intermediates, including phenylpyruvate, l-phenylalanine, homogentisate, dopamine, etc., were significantly increased in sepsis. The ratio of the phenylalanine-to-tyrosine ratio is associated with immune activation, which decreases with effective antiretroviral therapy [24]. The catabolic pathway converting phenylalanine to phenylpyruvate was revealed to promote the neutrophil-evasive state in an *Acinetobacter baumannii* study [25]. An excess of l-phenylalanine was found to inhibit protein synthesis, which had an effect on antibody production [26]. Moreover, dopamine inhibits the release of cytokines to modulate cellular immune function and suppress systemic inflammation via electroacupuncture [27, 28]. Treatments with removing various harmful and excess metabolites from body to purify the blood and acid–base have been widely used, like dialysis and artificial liver support system. And targeting metabolism is an effective way in cancer treatments, such as that with methotrexate, glycolytic inhibitor. Optimizing concentrations of metabolites might effectively regulate hyperinflammatory responses of sepsis, which further support the hypothesis that they change the mechanisms underlying poor outcomes in sepsis and thereby present potential therapeutic targets. And the effects of modulating metabolism on systemic inflammatory response in sepsis would be validated ex vivo in the further study.

Multiomics analysis of metabolomics and proteomics datasets based on the same biological samples was applied in this study. Integration of multiple omics data might provide novel biological information that is not revealed in a single dataset. In addition, identifying the correlation of variables across different methodologically and biologically distinct datasets could help to construct a reliable network with mutual functional validation, providing the biological process from the encoding gene to the final metabolic products and revealing key drivers associated with disease pathological mechanism [29]. And these features might serve as biomarker candidates for diagnosis and therapeutic targets. Sixteen proteins, including FIBA, SAA2, A2GL, AACT, CRP, LBP, SAA1, and S10A9, were identified as potential biomarkers in sepsis through integrative multiomics analysis, which is consistent with prior evidence [13]. These results illustrated that the multiomics analysis results had high reliability. Functional enrichment analysis of proteins showed the characteristic biological functions of sepsis, such as the acute inflammatory response and Toll-like receptor signalling pathway. The results showed that the downstream metabolites of proteins were mainly involved in amino acid metabolism, including phenylalanine and arginine biosynthesis, further illustrating that the main metabolic disorder in sepsis is amino acid imbalance. The coexpression network analysis revealed that module cyan was significantly correlated with SAE, with the eigenmetabolite value decreasing during disease development. Module cyan was enriched the pentose phosphate pathway, which regulates the production of NADPH and promotes antioxidant defence [30]. Studies have revealed dysregulation of the pentose phosphate pathway in Alzheimer’s disease, which is an incurable neurodegenerative disease [31]. Module yellow was highly positively associated with sepsis-AKI and mostly contained benzenoids and organic acids. Due to the limited number of metabolites, no specific metabolic pathways were significantly enriched in module yellow. Further information on patients with SAE and sepsis-AKI should be clarified in future studies.

The metabolites for discriminating patients with sepsis were identified in this study based on differential expression analysis and OPLS-DA. In addition to amino acids, some glycerophospholipids, fatty acids, beta hydroxy acids and carbonyl compounds had statistical significance, high fold changes and VIP scores in the comparison between sepsis and NC subjects. These metabolites could discriminate sepsis patients from NC subjects with high accuracy and sensitivity due to their specific concentration in sepsis patients, indicating they are biomarker candidates in the diagnosis of sepsis. The results revealed that the level of glycerophospholipids decreased in sepsis. Glycerophospholipids are a vital part of biomembranes and participate in signal transduction and the immune response. The production of glycerophospholipids regulates phagocytosis and platelet degranulation [32]. Crucial fatty acids are upregulated in sepsis and have protective effects on epithelial cells [33]. The increased level of fatty acids probably has anti-inflammatory functions.

## Conclusions

In summary, this study presents an integrative analysis of metabolomic and proteomic data based on the same biological samples from patients with sepsis and NC subjects. The characteristic proteins and metabolites identified formed a complex network to depict the crucial immunometabolism linked to sepsis. Amino acid-related pathways, including phenylalanine metabolism, tyrosine metabolism and tryptophan biosynthesis, were illustrated to be essential mechanisms of sepsis. The pentose phosphate pathway was revealed to have a potential effect on SAE. The changed metabolites might provide useful diagnostic and therapeutic methods for sepsis.

## Supplementary Information

**Additional file 1: Figure S1.** Quality control (QC) of experimental data. (A) Comparison of the total ion chromatogram (TIC) of QC samples in positive ion mode (left) and negative ion mode (right). (B) Principal component analysis (PCA) of the identified metabolites showing QC samples clustered together both in positive ion mode (left) and negative ion mode (right). **Figure S2.** Evaluation parameters of the OPLS-DA model in positive ion mode (left) and negative ion mode (right). **Figure S3.** Venn diagram showing the hub metabolites in positive ion mode (left) and negative ion mode (right). **Figure S4.** Cluster dendrogram and 16 metabolite coexpression modules defined by dendrogram branch cutting of all identified metabolites of patients with sepsis and normal controls.

**Additional file 2: Table S1.** Characteristics of enrolled patients with sepsis and NC subjects included in the derivation group.

**Additional file 3: Table S2.** Differentially expressed metabolites of the comparison between sepsis patients and NC subjects in the positive ion mode.

**Additional file 4: Table S3.** Differentially expressed metabolites of the comparison between sepsis patients and NC subjects in the negative ion mode.

## Data Availability

All the data generated and analyzed during this study are included in the manuscript and the additional materials.

## Abbreviations

WGCNA: Weighted gene coexpression network analysis
ICU: Intensive care unit
SOFA: Sequential organ failure assessment
NC: Normal control
UPLC-Q-TOF/MS: Ultrahigh-performance liquid chromatography with quadrupole time-of-flight mass spectrometry
HILIC: Hydrophilic interaction liquid chromatography
ESI: Electrospray ionization
RPLC: Reversed phase liquid chromatography
KEGG: Kyoto Encyclopedia of Genes and Genomes
DIABLO: Data Integration Analysis for Biomarker Discovery Using Latent cOmponents
SD: Standard deviation
IQR: Interquartile range
DEM: Differentially expressed metabolites
FC: Fold change
PCA: Principal component analysis
OPLS-DA: Orthogonal partial least-squares discrimination analysis
APACHE II: Acute physiology and chronic health evaluation II
QC: Quality control
VIP: Variable importance in the projection
AUROC: Area under the receiver operating characteristic curve
SAE: Sepsis-associated encephalopathy
Sepsis-AKI: Sepsis-associated acute kidney injury

## References

[1] Singer M, Deutschman CS, Seymour CW, Shankar-Hari M, Annane D, Bauer M, Bellomo R, Bernard GR, Chiche JD, Coopersmith CM (2016). The third international consensus definitions for sepsis and septic shock (Sepsis-3). JAMA.

[2] Rudd KE, Johnson SC, Agesa KM, Shackelford KA, Tsoi D, Kievlan DR, Colombara DV, Ikuta KS, Kissoon N, Finfer S (2020). Global, regional, and national sepsis incidence and mortality, 1990–2017: analysis for the Global Burden of Disease Study. Lancet.

[3] Reinhart K, Daniels R, Kissoon N, Machado FR, Schachter RD, Finfer S (2017). Recognizing sepsis as a global health priority—a who resolution. N Engl J Med.

[4] Patti GJ, Yanes O, Siuzdak G (2012). Innovation: metabolomics: the apogee of the omics trilogy. Nat Rev Mol Cell Biol.

[5] Johnson CH, Ivanisevic J, Siuzdak G (2016). Metabolomics: beyond biomarkers and towards mechanisms. Nat Rev Mol Cell Biol.

[6] Lee J, Banerjee D (2020). Metabolomics and the microbiome as biomarkers in sepsis. Crit Care Clin.

[7] Blaise BJ, Gouel-Cheron A, Floccard B, Monneret G, Allaouchiche B (2013). Metabolic phenotyping of traumatized patients reveals a susceptibility to sepsis. Anal Chem.

[8] Mickiewicz B, Duggan GE, Winston BW, Doig C, Kubes P, Vogel HJ, Alberta Sepsis N (2014). Metabolic profiling of serum samples by 1H nuclear magnetic resonance spectroscopy as a potential diagnostic approach for septic shock. Crit Care Med.

[9] Pei G, Chen L, Zhang W (2017). WGCNA application to proteomic and metabolomic data analysis. Methods Enzymol.

[10] Karczewski KJ, Snyder MP (2018). Integrative omics for health and disease. Nat Rev Genet.

[11] Luo D, Deng T, Yuan W, Deng H, Jin M (2017). Plasma metabolomic study in Chinese patients with wet age-related macular degeneration. BMC Ophthalmol.

[12] Gu Z, Li L, Tang S, Liu C, Fu X, Shi Z, Mao H (2018). Metabolomics reveals that crossbred dairy buffaloes are more thermotolerant than Holstein cows under chronic heat stress. J Agric Food Chem.

[13] Liang X, Wu T, Chen Q, Jiang J, Jiang Y, Ruan Y, Zhang H, Zhang S, Zhang C, Chen P (2021). Serum proteomics reveals disorder of lipoprotein metabolism in sepsis. Life Sci Alliance.

[14] Langfelder P, Horvath S (2008). WGCNA: an R package for weighted correlation network analysis. BMC Bioinform.

[15] Singh A, Shannon CP, Gautier B, Rohart F, Vacher M, Tebbutt SJ, Le Cao KA (2019). DIABLO: an integrative approach for identifying key molecular drivers from multi-omics assays. Bioinformatics.

[16] Rohart F, Gautier B, Singh A, Le Cao KA (2017). mixOmics: an R package for ‘omics feature selection and multiple data integration. PLoS Comput Biol.

[17] Rhodes A, Evans LE, Alhazzani W, Levy MM, Antonelli M, Ferrer R, Kumar A, Sevransky JE, Sprung CL, Nunnally ME (2017). Surviving sepsis campaign: international guidelines for management of sepsis and septic shock: 2016. Intensive Care Med.

[18] Schmerler D, Neugebauer S, Ludewig K, Bremer-Streck S, Brunkhorst FM, Kiehntopf M (2012). Targeted metabolomics for discrimination of systemic inflammatory disorders in critically ill patients. J Lipid Res.

[19] Xiong L, Teng JL, Botelho MG, Lo RC, Lau SK, Woo PC (2016). Arginine metabolism in bacterial pathogenesis and cancer therapy. Int J Mol Sci.

[20] Li P, Yin YL, Li D, Kim SW, Wu G (2007). Amino acids and immune function. Br J Nutr.

[21] Basler T, Meier-Hellmann A, Bredle D, Reinhart K (2002). Amino acid imbalance early in septic encephalopathy. Intensive Care Med.

[22] Ravindran R, Loebbermann J, Nakaya HI, Khan N, Ma H, Gama L, Machiah DK, Lawson B, Hakimpour P, Wang YC (2016). The amino acid sensor GCN2 controls gut inflammation by inhibiting inflammasome activation. Nature.

[23] McGaha TL, Huang L, Lemos H, Metz R, Mautino M, Prendergast GC, Mellor AL (2012). Amino acid catabolism: a pivotal regulator of innate and adaptive immunity. Immunol Rev.

[24] Zangerle R, Kurz K, Neurauter G, Kitchen M, Sarcletti M, Fuchs D (2010). Increased blood phenylalanine to tyrosine ratio in HIV-1 infection and correction following effective antiretroviral therapy. Brain Behav Immun.

[25] Rodman N, Martinez J, Fung S, Nakanouchi J, Myers AL, Harris CM, Dang E, Fernandez JS, Liu C, Mendoza AM (2019). Human pleural fluid elicits pyruvate and phenylalanine metabolism in *Acinetobacter baumannii* to enhance cytotoxicity and immune evasion. Front Microbiol.

[26] Ryan WL, Carver MJ (1964). Inhibition of antibody synthesis by l-phenylalanine. Science.

[27] Oberbeck R, Schmitz D, Wilsenack K, Schuler M, Husain B, Schedlowski M, Exton MS (2006). Dopamine affects cellular immune functions during polymicrobial sepsis. Intensive Care Med.

[28] Torres-Rosas R, Yehia G, Pena G, Mishra P, del Rocio T-B, Moreno-Eutimio MA, Arriaga-Pizano LA, Isibasi A, Ulloa L (2014). Dopamine mediates vagal modulation of the immune system by electroacupuncture. Nat Med.

[29] Lee AH, Shannon CP, Amenyogbe N, Bennike TB, Diray-Arce J, Idoko OT, Gill EE, Ben-Othman R, Pomat WS, van Haren SD (2019). Dynamic molecular changes during the first week of human life follow a robust developmental trajectory. Nat Commun.

[30] Wu S, Wang H, Li Y, Xie Y, Huang C, Zhao H, Miyagishi M, Kasim V (2018). Transcription factor YY1 promotes cell proliferation by directly activating the pentose phosphate pathway. Cancer Res.

[31] Demarest TG, Varma VR, Estrada D, Babbar M, Basu S, Mahajan UV, Moaddel R, Croteau DL, Thambisetty M, Mattson MP, Bohr VA (2020). Biological sex and DNA repair deficiency drive Alzheimer’s disease via systemic metabolic remodeling and brain mitochondrial dysfunction. Acta Neuropathol.

[32] Shen B, Yi X, Sun Y, Bi X, Du J, Zhang C, Quan S, Zhang F, Sun R, Qian L (2020). Proteomic and metabolomic characterization of COVID-19 patient sera. Cell.

[33] Li M, van Esch B, Wagenaar GTM, Garssen J, Folkerts G, Henricks PAJ (2018). Pro- and anti-inflammatory effects of short chain fatty acids on immune and endothelial cells. Eur J Pharmacol.

