# Correction to: Integrative analysis of metabolomics and proteomics reveals amino acid metabolism disorder in sepsis

**DOI:** 10.1186/s12967-022-03548-8

**Published:** 2022-08-15

**Authors:** Qi Chen, Xi Liang, Tianzhou Wu, Jing Jiang, Yongpo Jiang, Sheng Zhang, Yanyun Ruan, Huaping Zhang, Chao Zhang, Peng Chen, Yuhang Lv, Jiaojiao Xin, Dongyan Shi, Xin Chen, Jun Li, Yinghe Xu

**Affiliations:** 1 Precision Medicine Center, Taizhou Central Hospital (Taizhou University Hospital), Taizhou, China; 2 State Key Laboratory for Diagnosis and Treatment of Infectious Diseases, National Clinical Research Center for Infectious Diseases, National Medical Center for Infectious Diseases, Collaborative Innovation Center for Diagnosis and Treatment of Infectious Diseases, The First Affiliated Hospital, Zhejiang University School of Medicine, Hangzhou, China; 3 Key Laboratory for Critical Care Medicine, Multidisciplinary Collaborative Innovation Team for Diagnosis and Treatment of Critical Care Medicine, Taizhou Central Hospital (Taizhou University Hospital), Taizhou, China; 4 Department of Intensive Care Unit, Taizhou Hospital of Zhejiang Province, Taizhou Central Hospital (Taizhou University Hospital), Wenzhou Medical University, 999 Donghai Avenue, Taizhou, 318000 China; 5 Department of Intensive Care Unit, Taizhou Enze Medical Center (Group) Enze Hospital, Taizhou, China; 6 Institute of Pharmaceutical Biotechnology and the First Affiliated Hospital Department of Radiation Oncology, Zhejiang University School of Medicine, Hangzhou, 310058 China; 7 Joint Institute for Genetics and Genome Medicine Between, Zhejiang University and University of Toronto, Zhejiang University, Hangzhou, China

## Correction to: Journal of Translational Medicine (2022) 20:123 10.1186/s12967-022-03320-y

Following publication of the original article [1], we have been notified that one of the authors, Tianzhou Wu, was not marked as one of the authors who contributed equally to the work. Also, authors Xin Chen and Jun Li should be mentioned as corresponding authors.

We have also been notified that affiliations 2 and 3 should be modified. They should be as follows:

- 2 State Key Laboratory for Diagnosis and Treatment of Infectious Diseases, National Clinical Research Center for Infectious Diseases, National Medical Center for Infectious Diseases, Collaborative Innovation Center for Diagnosis and Treatment of Infectious Diseases, The First Affiliated Hospital, Zhejiang University School of Medicine, Hangzhou, China.
- 3 Key Laboratory for Critical Care Medicine, Multidisciplinary Collaborative Innovation Team for Diagnosis and Treatment of Critical Care Medicine, Taizhou Central Hospital (Taizhou University Hospital), Taizhou, China.
