# Supplementary material for: Integrative analysis of metabolomics and proteomics reveals amino acid metabolism disorder in sepsis
Source: J Transl Med. 2022 Mar 14;20:123. doi: 10.1186/s12967-022-03320-y (PMC8919526; doi:10.1186/s12967-022-03320-y)
Supplement: Supplementary file 1 — Additional file 1: Figure S1. Quality control (QC) of experimental data. (A) Comparison of the total ion chromatogram (TIC) of QC samples in positive ion mode (left) and negative ion mode (right). (B) Principal component analysis (PCA) of the identified metabolites showing QC samples clustered together both in positive ion mode (left) and negative ion mode (right). Figure S2. Evaluation parameters of the OPLS-DA model in positive ion mode (left) and negative ion mode (right). Figure S3. Venn diagram showing the hub metabolites in positive ion mode (left) and negative ion mode (right). Figure S4. Cluster dendrogram and 16 metabolite coexpression modules defined by dendrogram branch cutting of all identified metabolites of patients with sepsis and normal controls. [file 12967_2022_3320_MOESM1_ESM.docx]

Additional file 1

***Contents***

- Additional file 1: Figure S1
- Additional file 1: Figure S2
- Additional file 1: Figure S3
- Additional file 1: Figure S4
- Additional file 1: Table S1
- Additional file 1: Table S2
- Additional file 1: Table S3

**
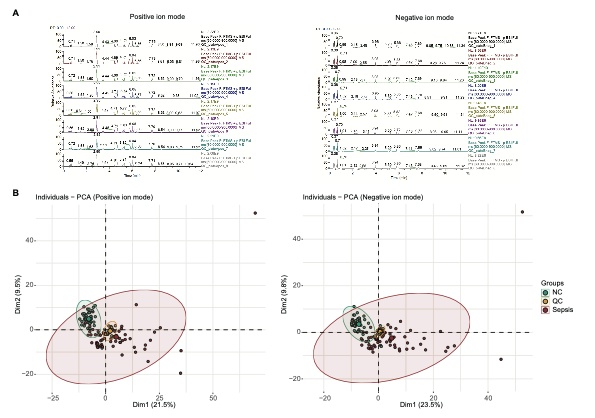
**

**Additional file 1: Figure S1 Quality control (QC) of experimental data.** (A) Comparison of the total ion chromatogram (TIC) of QC samples in positive ion mode (left) and negative ion mode (right). (B) Principal component analysis (PCA) of the identified metabolites showing QC samples clustered together both in positive ion mode (left) and negative ion mode (right).

**Additional file 1: Figure S2 Evaluation parameters of the OPLS-DA model in positive ion mode (left) and negative ion mode (right).**

**
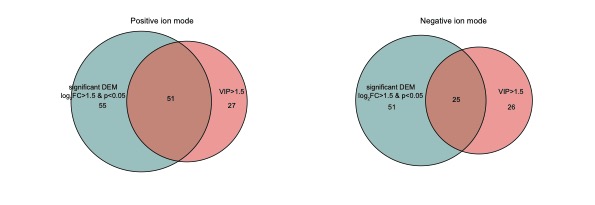
**

**Additional file 1: Figure S3 Venn diagram showing the hub metabolites in positive ion mode (left) and negative ion mode (right).**

**Additional file 1: Figure S4** **Cluster dendrogram and 16 metabolite coexpression modules defined by dendrogram branch cutting of all identified metabolites of patients with sepsis and normal controls.**
