# Supplementary material for: Integrative analysis of metabolomics and proteomics reveals amino acid metabolism disorder in sepsis
Source: J Transl Med. 2022 Mar 14;20:123. doi: 10.1186/s12967-022-03320-y (PMC8919526; doi:10.1186/s12967-022-03320-y)
Supplement: Supplementary file 2 — Additional file 2: Table S1. Characteristics of enrolled patients with sepsis and NC subjects included in the derivation group. [file 12967_2022_3320_MOESM2_ESM.docx]

**Table S1** Characteristics of enrolled patients with sepsis and NC subjects included in the derivation group.

|  | Sepsis | NC | P value |
| --- | --- | --- | --- |
| n | 42 | 30 |  |
| Male (%) | 26 (61.9%) | 18 (60.0%) | 1.000 |
| Age (years) | 71.5 [61.0, 78.0] | 68.00 [65.00, 71.25] | 0.706 |
| **Laboratory Data** |  |  |  |
| Mean arterial pressure (mm Hg) | 79.7 [74.0, 89.0] | NA | NA |
| White blood cell count (10^9^/L) | 14.3 [10.1, 21.1] | 5.40 [4.53, 6.50] | <0.001 |
| Haemoglobin (g/L) | 119.0 [101.5, 129.5] | 147.00 [138.25, 159.75] | <0.001 |
| Haematocrit (%) | 35.8 [31.5, 38.8] | 43.90 [39.50, 47.35] | <0.001 |
| Platelet count (10^9^/L) | 136.0 [60.7, 209.5] | 226.50 [192.50, 237.25] | <0.001 |
| Albumin (g/dL) | 27.0 [24.4, 30.9] | 45.40 [43.70, 46.20] | <0.001 |
| Aspartate aminotransferase (U/L) | 45.0 [21.2, 89.5] | 20.00 [18.00, 22.25] | <0.001 |
| Alanine aminotransferase (U/L) | 29.5 [14.5, 61.5] | 19.00 [14.75, 27.00] | 0.042 |
| Total bilirubin (μmol/L) | 16.5 [9.0, 25.1] | 16.60 [12.15, 19.08] | 0.805 |
| Creatinine (μmol/l) | 133.0 [98.7, 226.0] | 74.00 [57.50, 79.50] | <0.001 |
| INR | 1.2 [1.1, 1.3] | NA | NA |
| **Infection** |  | NA | NA |
| Gram-positive bacteria (%) | 4 (9.5%) |  |  |
| Gram-negative bacteria (%) | 18 (42.9%) |  |  |
| Viral (%) | 1 (2.4%) |  |  |
| Other (%) | 19 (45.2%) |  |  |
| **CRRT** | 8 (13.6%) | NA | NA |
| **Vasopressors** |  | NA | NA |
| 0 (%) | 18 (42.9%) |  |  |
| 1 (%) | 20 (47.6%) |  |  |
| NA (%) | 4 (9.5%) |  |  |
| **Mechanical ventilation** |  | NA | NA |
| 0 (%) | 21 (50.0%) |  |  |
| 1 (%) | 15 (35.7%) |  |  |
| NA (%) | 6 (14.3%) |  |  |
| **Severity at time of admission to ICU** |  |  |  |
| SOFA | 6.0 [4.0, 9.0] | NA | NA |
| APACHE II | 19.0 [10.2, 22.7] | NA | NA |
| **Mortality** |  |  |  |
| 28-day | 6 (14.3%) | NA | NA |
| 90-day | 6 (14.3%) | NA | NA |

Data are expressed as the mean ± SD, median (IQR) or number of patients (percentages). Continuous variables were compared by using Student’s t test and the Mann–Whitney U test, and categorical variables were compared by using the χ2 or Fisher’s exact test between the derivation and validation groups.

APACHE II=Acute Physiology and Chronic Health Evaluation II. SOFA=Sequential Organ Failure Assessment on day of sampling.
